# Vitamin D Metabolism and the Risk of Renal Stones: A Focus on PHPT

**DOI:** 10.3390/metabo15100639

**Published:** 2025-09-24

**Authors:** Elena Castellano, Federica Saponaro

**Affiliations:** 1 Department of Endocrinology, Diabetes and Metabolism, Santa Croce and Carle Hospital, 12100 Cuneo, Italy; 2 Department of Surgical, Medical and Molecular Pathology and Critical Care Medicine, University of Pisa, Via Roma 56, 56126 Pisa, Italy; federica.saponaro@unipi.it

**Keywords:** vitamin D, primary hyperparathyroidism, renal stones

## Abstract

Primary hyperparathyroidism is nowadays a common endocrine disorder. Over time, the clinical manifestation has shifted from symptomatic cases to mostly asymptomatic diagnoses. Despite this, nephrolithiasis remains significant, often presenting as bilateral and recurrent, with the literature reporting prevalence rates of up to 40%. The nephrolithiasis pathogenesis in PHPT is multifactorial and not fully understood. While elevated PTH increases urinary calcium load, additional urinary abnormalities and demographic factors, including age and sex, influence the risk. Vitamin D status has also been explored as a possible contributor to stone formation both in the general population and in PHPT patients. The relationship between serum 25OHD levels and nephrolithiasis remains unclear, and the impact of vitamin D supplementation on stone risk in PHPT is still under investigation. The relationship between vitamin D status, supplementation and renal stones in PHPT is explored in the present review.

## 1. Introduction

Primary hyperparathyroidism (PHPT) currently represents the third most common endocrine disorder, with an estimated prevalence of approximately 0.4% in the general population and, rising significantly with age, particularly among postmenopausal women [1].

In recent decades, the clinical presentation of PHPT in Western Countries has progressively moved from overt symptomatic disease, typically characterized by bone and renal involvement, to predominantly asymptomatic cases, driven by the widespread routine testing of serum calcium levels and bone density screening [1,2]. Nevertheless, nephrolithiasis remains a clinically relevant manifestation, frequently presenting as bilateral and recurrent disease [3,4,5]. The reported prevalence of nephrolithiasis among PHPT patients varies in data in the literature, with rates of up to 40% depending on study design and the imaging modality used [6,7]. Ultrasonography (US) is the most commonly used modality for renal stone screening. Routine renal imaging is recommended by current clinical guidelines and allows the detection of asymptomatic nephrolithiasis, so-called “silent nephrolithiasis” [1,8]. Asymptomatic renal stones occur more frequently in PHPT patients than in the general population [9] and are typically smaller than symptomatic ones [10]. Renal stones in PHPT are predominantly composed of calcium oxalate and calcium phosphate, in accordance with the underlying metabolic alterations induced by PTH excess [4,11,12].

Although the pathogenesis of nephrolithiasis in PHPT remains incompletely understood, it is likely to be multifactorial [12,13]. Elevated PTH levels enhance renal calcium reabsorption, bone resorption, and intestinal calcium absorption, increasing urinary calcium load and contributing to stone formation [12]. Consistently, elevated urinary calcium is included among the surgical criteria for asymptomatic PHPT, reflecting its potential role in stone risk predisposition [1,13].

Nevertheless, the association between hypercalciuria and stone formation in PHPT is not entirely linear. Not all hypercalciuric PHPT patients develop renal stones and individuals with nephrolithiasis do not routinely have higher urinary calcium levels compared to their stone-free counterparts. Additional contributing mechanisms of nephrolithiasis in PHPT patients include other urinary abnormalities such as hypocitraturia, altered phosphate handling, hypomagnesuria and alterations in urinary pH [13]. Moreover, age and sex strongly modulate lithogenic risk in PHPT, with younger individuals and males being at higher risk [4,10]. Younger patients typically have greater renal functional mass and higher renal 1α-hydroxylase activity, which leads to increased circulating 1,25-dihydroxyvitamin D and increased intestinal calcium absorption, further increasing urinary calcium excretion [14,15]. The higher risk in men is generally attributed to various factors, such as dietary and lifestyle differences, as well as genetic and hormonal causes [15,16]. These demographic trends mirror those observed in the general population, where the lifetime prevalence of nephrolithiasis is approximately 13% in men and 7% in women [16].

Parathyroidectomy (PTX) reduces serum calcium and PTH levels, significantly lowering urinary calcium excretion and the recurrence rate of renal stones in PHPT patients, even though it does not completely eliminate the risk of renal stone recurrence [17,18].

Vitamin D status has also been explored as a possible contributor to stone formation both in the general population [19] and in PHPT patients [20]. However,, the relationship between serum 25OHD levels and nephrolithiasis remains unclear, and the impact of vitamin D supplementation on stone risk in PHPT is still under investigation. While vitamin D supplementation is essential for bone health in PHPT patients with vitamin D deficit [1,2], excessive intake may precipitate hypercalcemia and hypercalciuria, theoretically increasing the lithogenic risk.

The relationship between vitamin D status, supplementation and renal stones in PHPT is explored in the present review.

## 2. Vitamin D and Renal Stones

Vitamin D plays a crucial role in various physiological processes beyond just bone health [21]. Conversely, kidney stones are a common urinary disorder influenced by factors such as diet, lifestyle, and genetics [22]. As already mentioned, the complex relationship between vitamin D and kidney stones requires further investigation, particularly in the context of PHPT, a clinical condition in which calcium homeostasis is profoundly affected and the reciprocal connection between PTH and vitamin D is even more complex, nuanced, and partially still to be clarified.

To summarize the knowledge regarding this issue we primarily recapitulate the role of vitamin D in the maintenance of calcium/phosphorus homeostasis and the possible mechanisms linking hypovitaminosis D with the prevalence of renal stones.

### 2.1. Vitamin D Role in Calcium/Phosphorus Homeostasis

Vitamin D exists in two different forms: vitamin D2 (ergocalciferol) which has a minor role in humans and is primarily derived from plant-based foods, vitamin D3 (cholecalciferol), synthesized in the skin from 7-dehydrocholesterol (7DHC), an intermediate in cholesterol production, through exposure to ultraviolet B (UVB) radiation. Vitamin D undergoes two hydroxylation processes: the first occurs in the liver, resulting in the formation of 25-hydroxyvitamin D3 (25(OH)D3), followed by the second step in the kidneys, or alternatively in other tissues and organs, as was shown more recently. This latter step leads to the production of 1,25-dihydroxyvitamin D (1,25(OH)2D), the hormone’s active form, which plays a vital role in sustaining bone homeostasis and calcium metabolism, along with various protective effects outside of the skeletal system [23,24].

Vitamin D exhibits a complex, multistage metabolism affecting both skeletal and numerous extra-skeletal targets [24], influencing approximately 3% of the entire genome from zebrafish to humans, either directly or indirectly. The widespread distribution of intracellular vitamin D receptors (VDR) and the 1α-hydroxylase isoenzyme across various tissues highlights the hormone’s diverse functions.

Vitamin D is crucial for maintaining the body’s balance of calcium and phosphorus and significantly enhances the absorption of these essential minerals in the intestines. This enhancement is achieved through vitamin D’s binding to the vitamin D receptor (VDR) in intestinal cells, leading to physiological responses that increase the efficiency of calcium transport across the gastrointestinal epithelium via the TRPV6 transporter protein. In addition to its role in calcium absorption, vitamin D also facilitates phosphorus utilization within the body [25,26].

Furthermore, vitamin D regulates the secretion of hormones particularly PTH and fibroblast growth factor 23 (FGF23), which are critical for maintaining blood concentrations of calcium and phosphorus [27]. Parathyroid gland cells express the vitamin D receptor (VDR), which, when stimulated, decreases parathyroid hormone (PTH) production and secretion, inhibits cell growth, and increases calcium-sensing receptor expression [28]. This reduces circulating PTH levels, preventing overcorrection of low calcium, the major stimulus of PTH secretion. The PTH gene has a vitamin D response element that, when bound by the VDR-RXR complex, inhibits PTH transcription. Parathyroids also have the ability to convert circulating vitamin D to its active form via CYP27B1 [29]. The relationship between circulating and locally produced vitamin D in regulating PTH synthesis is complex, with mild vitamin D deficiency possibly leading to secondary hyperparathyroidism due to reduced active vitamin D production [30].

PTH itself controls vitamin D metabolism leading to the enhancement of renal production of 1,25 vitamin D through increasing Cyp27B1 expression and decreasing Cyp24 expression, in a finely tuned feedback and regulatory mechanism that becomes even more complex under pathological conditions, such as PHPT [31].

Vitamin D stimulates bone cells to produce fibroblast growth factor 23 (FGF23) and inhibits PHEX, an enzyme that deactivates FGF23. This results in increased FGF23 levels and phosphate excretion. The activity of FGF23 reduces active vitamin D levels by promoting its inactivation and decreasing its production in the kidneys. Moreover, FGF23 also inhibits vitamin D activation in non-renal tissues such as the parathyroid gland and placenta [32].

### 2.2. Vitamin D and Kidney Stones

The relationship between vitamin D and kidney stones has been frequently studied due to vitamin D’s involvement in calcium regulation. Excessive vitamin D can result in hypercalciuria, promoting the formation of calcium oxalate (CaOX) stones, primarily made up of calcium oxalate dihydrate (COD) crystals. Research findings on the association between 25-hydroxyvitamin D levels and the risk of kidney stone development have yielded inconsistent results, particularly in the context of normal serum vitamin D levels or vitamin D deficiency. Consequently, clinicians often hesitate to manage vitamin D deficiency in patients with urolithiasis.

Numerous observational studies and a limited number of clinical trials have investigated the link between serum vitamin D levels and the likelihood of kidney stones or evaluated the impact of vitamin D supplementation on stone formation while excluding patients with elevated serum 25-hydroxyvitamin D levels, as discussed later in this review.

Contrary to the established hypothesis that higher serum 25-hydroxyvitamin D levels promote kidney stone formation, vitamin D deficiency may also contribute to the development or intensity of kidney stones, suggesting that the relationship between vitamin D and the formation of kidney stones is likely more complex and follows a U-shaped curve. Recent research has highlighted a significant prevalence of vitamin D deficiency among stone formers, with even higher rates in comparison to non-stone formers. In a 2021 prospective case–control study of 200 patients divided into patients with (n = 100) or without (n = 100) urolithiasis, Dholakia et al. demonstrated that the prevalence of vitamin D deficiency was significantly higher in the first group compared to the second (95% vs. 57%),and secondary hyperparathyroidism was present in 71% of patients due to hypovitaminosis D, a prevalent contributor to stone formation [33].

Similar results have been found by Elkoushy et al. in a retrospective study on 101 patients with nephrolithiasis. Out of 101 patients, 80% were found to have vitamin D insufficiency and hyperparathyroidism was present in 26% of the patients, with 91% of these cases attributed to hypovitaminosis. They described common urinary abnormalities in patients with hypovitaminosis D including suboptimal urine volume (45%), hypocitraturia (24%), hypocalciuria (33%), hypercalciuria (20%), hyperuricosuria (16%), cystinuria (5%), and hyperoxaluria (7.2%), which are all risk factors for stone formation and deposition [34]. Another study by Pipili et al. examined vitamin D levels in 236 patients with recurrent kidney stones and found, that calcium oxalate stones were the most common type. One-third of participants had vitamin D insufficiency, while a quarter showed high PTH levels despite normal calcium. Low vitamin D levels were strongly linked to higher PTH and stone formers with hypercalciuria had higher vitamin D levels than those with normal urinary calcium [35].

A wider Italian cohort was evaluated by Ticinesi et al. in a case–control study involving 884 patients with idiopathic calcium nephrolithiasis (ICN) and 967 controls. Following a fully adjusted conditional logistic regression analysis conducted on propensity-matched cohorts (442 stone formers and 442 controls), a significant association was found between vitamin D deficiency and the likelihood of nephrolithiasis, with an estimated odds ratio of 2.29 (95% confidence interval: 1.74–3.02, *p* < 0.001) [36].

However, a number of older studies produced results that did not align with the previous conclusions. For example, research conducted by Netelenbos et al. and Tang et al. in Colorado, USA, indicated that levels of serum 25(OH)D were comparable between stone formers (SFs) and non-stone formers (non-SFs) [37,38]. Additionally, other investigations demonstrated that SFs exhibited significantly elevated levels of 1,25(OH)2D compared to their non-SF counterparts [39,40].

These differences in outcomes may stem from the fact that the studies took place in various countries, each with substantial differences in geographic, cultural, social, and economic contexts, as well as genetic characteristics of their populations. Moreover, we currently do not have detailed knowledge on the relationship between different metabolites of vitamin D in physiopathological conditions, an issue that will be faced by studies using HPLC-MS-MS to measure the vitamin D metabolome.

However, if this is the case, what are the possible explanations for the association between hypovitaminosis D and kidney stones? Some hypotheses have been proposed: one hypothesis proposes that secondary hyperparathyroidism in individuals with vitamin D deficiency may itself increase their risk of kidney stones.

Another potential explanation involves shared risk factors for both vitamin D deficiency and kidney stone formation, such as older age, female gender, low socioeconomic status, malnutrition, and obesity, with obesity being the only common factor. Dietary aspects, including low calcium intake, high phytate consumption, and insufficient animal protein, may also contribute to vitamin D deficiency [1,2]. Low calcium intake can raise PTH levels, potentially leading to vitamin D deficiency. Additionally, some studies theorize that vitamin D deficiency may be connected to calcium stones through its role in promoting oxidative stress and inflammation in renal tissue, as vitamin D influences inflammatory transcription factors and adhesion molecules linked to kidney stone formation. Furthermore, vitamin D deficiency might affect the renin-renin–angiotensin–aldosterone system (RAAS), increasing oxidative stress and reactive oxygen species production, thus elucidating its association with kidney stone development [1].

Moreover, latitudinal differences in vitamin D and calcium metabolism may influence the relationship between vitamin D deficiency and the likelihood of nephrolithiasis. Research on vitamin D and calcium metabolism among high-latitude populations has primarily focused on the Inuit of Canada and Greenland. Sellers et al. [41] reported that the Inuit require less vitamin D due to their lower calcium requirements. Additionally, despite low blood calcium levels, they excrete excess calcium rapidly and absorb it more efficiently in their intestines. Rejnmark et al. [42] identified two unique physiological traits: a lower calcium-regulated parathyroid hormone release set-point and a heightened conversion rate of vitamin D to its active form.

These differences in vitamin D and calcium metabolism are probably adaptations to an inability to produce vitamin D in the skin during most of the year [43]. To date, no evidence has been reported on the influence of vitamin D supplementation at different latitudes on the risk of kidney stones in PHPT.

### 2.3. Vitamin D Deficiency, Hypercalciuria and Nephrolithiasis in PHPT

In the literature, it has been observed that patients with PHPT tend to have a higher prevalence of vitamin D deficiency compared to healthy individuals, and this deficiency may be linked to poorer disease outcomes. In Tassone et al’s observational study, vitamin D status was assessed in a cohort including 206 patients with PHPT at diagnosis compared to 113 age- and sex-matched healthy blood donors using plasma 25OHD levels. The findings indicated that 36.4% of PHPT patients had vitamin D deficiency, with significantly more prevalent hypovitaminosis D in PHPT patients than in healthy controls [44]. Raef et al.’s study explored the potential link between vitamin D deficiency and the severity of PHPT, analyzing 49 patients who underwent PTX. Their findings suggested that lower vitamin D levels correlate with more severe bone disease, larger parathyroid tumors, and increased biochemical markers of PHPT severity, suggesting that vitamin D deficiency may exacerbate both bone health issues and tumor growth in affected individuals [45].

In an Italian study by Viccica et al. the link between serum 25(OH)D levels and clinical characteristics was assessed, The authors evaluated 215 Italian women with PHPT who were not on vitamin D supplements. In a multivariate regression analysis, serum 25(OH)D was significantly correlated with age (r = −0.18; *p* = 0.005), BMI (r = −0.23; *p* = 0.049), serum PTH (r = −0.01; *p* = 0.023), BSAP (r = −0.01; *p* = 0.023), and eGFR (r = −0.09; *p* = 0.001), but not with R-BMD. Serum 25(OH)D levels were notably greater in patients with nephrolithiasis compared to those without (18.5 ± 8.8 ng/mL vs. 15.6 ± 8.0 ng/mL; *p* = 0.029); furthermore, the average 24-hour urinary calcium excretion was significantly higher in individuals with nephrolithiasis than in those without [46].

On the other hand, some studies revealed no association between hypovitaminosis D in PHPT and nephrolithiasis, suggesting that this issue is still debated and need more evidence. Indeed, Walker et al. showed that lower levels of 25OHD were correlated with certain measures of PHPT severity, including PTH and serum phosphate, but not with all measures, such as serum and urine calcium. A comparison of individuals with deficient (<20 ng/mL), insufficient (20–29 ng/mL), and adequate (≥30 ng/mL) levels of 25OHD indicated that those with lower levels experienced more severe PHPT, however, there were no significant differences observed in terms of nephrolithiasis and urinary calcium [20]. Consistently, a recent study published in 2022 on 128 patients with PHPT confirmed hypovitaminosis in a majority of patients (51.6%), but vitamin D deficiency was not associated with nephrolithiasis [47].

The relationship between vitamin D deficiency and nephrolithiasis in PHPT may be more complex than it appears. There may be a direct contribution to the development of nephrolithiasis; however, an indirect effect should also be considered. Hypovitaminosis D is prevalent in these patients and can obscure the anticipated hypercalcamia, thereby delaying the diagnosis of PHPT. Furthermore, a longer history of hypercalcaemic PHPT may exacerbate the renal phenotype, suggesting a multifaceted interplay between vitamin D levels and renal complications in this condition [48].

## 3. Vitamin D Supplementation and Renal Stones in PHPT

Historically, vitamin D supplementation in patients with PHPT has raised significant clinical concerns due to its potential to enhance intestinal calcium absorption. This physiological mechanism could consequently lead to elevated renal calcium excretion, increasing lithogenic potential and the risk of nephrolithiasis. Such apprehensions were notably discussed in early studies, including a critical viewpoint by Zaharani and Levine published in 1997 [49], suggesting caution and recommending avoidance of vitamin D supplementation in PHPT patients pending robust clinical evidence.

Overall, subsequent clinical investigations into vitamin D supplementation among PHPT patients with insufficient 25OHD levels have demonstrated beneficial outcomes, reduced PTH concentrations and stabilization of BMD, without significant short-term alterations in serum or urinary calcium [50]. However, these reassuring results are predominantly derived from short-term studies conducted in patients with mild or asymptomatic PHPT, which limits their generalizability.

While existing research has predominantly examined biochemical endpoints and bone metabolism, renal outcomes, and nephrolithiasis in particular, have received less attention. Although limited data exist on renal parameters, such as urinary calcium excretion and estimated glomerular filtration rate, data addressing the formation or progression of renal stones remains limited.

Kantorovich [51] evaluated vitamin D supplementation effects in five osteoporotic PHPT patients, observing short-term gains in BMD despite persistent inappropriate PTH secretion. Although hypercalcemia did not develop, frank hypercalciuria was induced in three previously normocalciuric patients. The short follow-up and the early surgical intervention precluded observation regarding nephrolithiasis development.

In a subsequent study, Grey [52] reported biochemical outcomes over 12 months in 21 vitamin D-deficient PHPT patients undergoing vitamin D repletion. Their data indicated no exacerbation of hypercalcemia, alongside reduced PTH and bone turnover markers. However, a subset of these patients exhibited increased urinary calcium excretion, even though symptomatic nephrolithiasis was reported. The lack of systematic renal imaging assessment limits conclusions regarding subclinical stone formation.

In 2014, Shah et Coll. conducted a meta-analysis including 340 PHPT patients from 10 studies [53]. Their findings confirmed that vitamin D supplementation significantly increases serum 25OHD levels and reduces serum PTH levels, without significant differences from baseline for serum and urinary calcium levels. In this regard, Shah’s study ran counter to the previous negative evidence of renal stone development post vitamin D supplementation.

The randomized controlled trial by Rolighed et al. [54] assessed the safety and efficacy of high-dose vitamin D supplementation (2800 IU/day) in 46 PHPT patients, administered before and after parathyroidectomy. The trial demonstrated decreased bone resorption markers and improved, BMD but again lacked routine renal imaging or systematic urinary calcium assessment, highlighting ongoing gaps in renal risk evaluation. A limitation across the available literature is the absence of comprehensive renal imaging and meticulous urinary calcium assessment both pre- and post-intervention, substantially limiting interpretations regarding subclinical nephrolithiasis. This aspect could be particularly relevant given the notable prevalence of silent renal stones in PHPT populations [9,10,55].

To date, no rigorous intervention study has systematically assessed vitamin D supplementation’s impact on nephrolithiasis incidence or risk in PHPT patients. Current evidence thus suggests that vitamin D repletion in PHPT patients with vitamin D deficiency does not elevate urinary calcium excretion nor clinically heighten nephrolithiasis risk, at least in short-term assessments.

It is likely that, as in the general population [56], the nephrolithiasis risk profile in PHPT patients is heterogeneous, influenced by genetic and environmental factors with distinct individual lithogenic pathways.

## 4. Other Predisposing Factors (Linked to Vitamin D Status?)

The formation of kidney stones is a multifactorial condition influenced by a variety of risk factors, including biological, genetic, and lifestyle components. Sex plays a significant role, as males are generally at higher risk than females, potentially due to differences in hormonal regulation and urinary composition. Genetic predisposition, such as variations in the calcium-sensing receptor (CasR), has been linked to altered calcium metabolism, further predisposing individuals, particularly those of African descent, to kidney stones [15].

Moreover, disturbances in bone metabolism can contribute to calcium imbalances, as conditions like osteoporosis may lead to increased calcium release into the bloodstream, elevating the risk of stone formation. Obesity and metabolic syndrome are also critical risk factors; they are associated with higher levels of uric acid and insulin resistance, which can both promote stone development. Finally, dietary and lifestyle factors, including high sodium intake, low fluid consumption, and diets rich in oxalate, can exacerbate the risk of lithogenesis by influencing urine composition and volume.

These same risk factors for kidney stone formation are also associated with vitamin D deficiency (hypovitaminosis D). In individuals with obesity and metabolic syndrome, the bioavailability of vitamin D can be reduced due to its sequestration in adipose tissue. This leads to lower serum levels of the active form of vitamin D, impairing intestinal calcium absorption and further contributing to disturbances in bone metabolism. Consequently, these individuals may experience secondary hyperparathyroidism, which can elevate calcium levels and increase the likelihood of kidney stones [57].

Furthermore, lifestyle factors such as a sedentary lifestyle, poor dietary habits, and inadequate sunlight exposure can exacerbate vitamin D deficiency. However, despite the plausible connections outlined between these risk factors and vitamin D deficiency in relation to kidney stones, current research does not provide definitive studies that directly correlate hypovitaminosis D with the incidence of kidney stones. Thus, while the theoretical framework is compelling, conclusive evidence is yet to be obtained.

Interventional studies on PHPT reveal no consensus on optimal vitamin supplementation. Active vitamin D metabolites like calcitriol are discouraged, favoring precursors to take advantage of the vitamin’s physiological effects [58].

Vitamin D3 would appear to be more effective than vitamin D2 in maintaining serum concentrations of 25-OHD due to its higher affinity for DBP [59].

To date, no evidence of a difference in the risk of renal stones using calcifediol or cholecalciferol in the general population or in PHPT subjects has been reported.

Moreover, cholecalciferol dosing regimens have not been evaluated as a predisposing factor for kidney stone formation in PHPT. This aspect has to be further explored in PHPT.

## 5. Conclusions

Current evidence indicates that vitamin D status may influence the risk of nephrolithiasis in both the general population and individuals with PHPT. Associations have been reported in the context of severe deficiency and excess of vitamin D, suggesting the involvement of both direct pathophysiological mechanisms and indirect pathways mediated by lithogenic or predisposing factors. Nevertheless, current data remain inconclusive and a definitive causal relationship has not yet been established.

Further large-scale, randomized controlled trials, ideally long-term and incorporating routine renal imaging, are imperative to definitively elucidate vitamin D supplementation’s effect on renal stone risk, particularly among PHPT patients managed conservatively over prolonged periods—a common practice in the clinical reality, especially among elderly or mildly symptomatic individuals who are often undertreated.

## Data Availability

Not applicable.
